# Solvent-Free Processing of i-P3HB Blends: Enhancing Processability and Mechanical Properties for Sustainable Applications

**DOI:** 10.3390/polym17162231

**Published:** 2025-08-16

**Authors:** Wael Almustafa, Sergiy Grishchuk, Michael Redel, Dirk W. Schubert, Gregor Grun

**Affiliations:** 1 Department of Applied Logistics and Polymer Sciences, Kaiserslautern University of Applied Science, Schoenstr. 11, 67659 Kaiserslautern, Germany; 2 Institute of Polymer Materials, Department of Materials Science, Faculty of Engineering, Friedrich-Alexander University Erlangen-Nürnberg (FAU), Martensstr. 7, 91058 Erlangen, Germany

**Keywords:** poly(3-hydroxybutyrate), blend, crystallinity, biodegradable, polyester

## Abstract

Poly(3-hydroxybutyrate) is a biobased and biodegradable polymer, produced via bacterial fermentation and characterized by an isotactic structure and mechanical properties similar to those of polyethylene and polypropylene. However, its brittleness—due to high crystallinity (~70%) and thermal degradation, starting at a temperature range of 180–190 °C near its melting point (175 °C)—makes its processing difficult and limits its applications. Most recent studies on modifying P3HB involved solution casting, typically using chloroform, which raises sustainability concerns. In this study blends of isotactic poly(3-hydroxybutyrate) (*i*-P3HB) with atactic poly(3-hydroxybutyrate) (*a*-P3HB) and poly(3-hydroxybutyrate-*co*-4-hydroxybutyrate) (P34HB) were prepared through solvent-free extrusion, and the thermal and mechanical properties of these blends were characterized. The obtained blends showed an extended processing window with reduced processing temperatures (150–160 °C), which were significantly lower than the onset of the decomposition temperature of *i*-P3HB, thereby avoiding thermal degradation. Furthermore, the crystallinity of these blends could be varied between 17 and 70%, depending on the polymer ratio, which allows for tailormade materials with tunable mechanical properties and an elongation at break up to 600%. Based on the results, the obtained blends in this study are promising candidates for various applications and processing techniques, such as injection molding, extrusion, and fiber spinning, offering a sustainable alternative to conventional plastics.

## 1. Introduction

Poly(3-hydroxybutyrate) (P3HB) is a biobased, biocompatible, and biodegradable polymer belonging to the polyhydroxyalkanoates (PHAs) family. It was first discovered and characterized by Maurice Lemoigne [1]. Poly(3-hydroxybutyrate) is primarily produced biotechnologically through bacterial fermentation in an isotactic form (*i*-P3HB) and exhibits a high crystallinity of around 70% [2]. The desirable properties, such as eco-friendliness, a resistance to organic solvents, and gas barrier capabilities and, at the same time, similar mechanical characteristics to those of polyethylene (PE) and polypropylene (PP) made P3HB an attractive candidate to replace those conventional plastics (Table 1) [3,4,5]. However, the high degree of crystallinity of neat *i*-P3HB also results in brittleness, and the onset of its thermal degradation, in terms of polymer chain scission, starting at a temperature range of 180–190 °C near its melting temperature (175 °C) complicates processing and hinders its application [6,7,8,9,10,11].

To address these challenges, improving the processability of *i*-P3HB and reducing its brittleness through chemical or physical modifications is crucial to facilitate its application in different sectors. For this approach, different strategies have been investigated in previous studies, such as the copolymerization with other hydroxy acids [13,14], the functionalization of *i*-P3HB with polyols followed by chain extension reactions [15,16,17], as well as blending *i*-P3HB with other polymers [18,19,20,21]. Among these strategies, blending *i*-P3HB with other polymers is particularly appealing, as it is industrially feasible without requiring significant investment [22,23,24].

Previous studies have explored blending *i*-P3HB with various polymers, including poly(ethylene oxide) [25,26], poly(vinyl alcohol) [27], poly(*ε*-caprolactone) [28,29], poly(butylene succinate) (PBS) [30,31], poly(ethylene succinate) (PES) [32], and poly[(butylene adipate)-*co*-terephthalate] (PBAT) [33]. However, these blends exhibited an incompatibility and phase separation, as indicated by thermal and mechanical analyses (e.g., distinct glass transition temperatures) [34].

An alternative strategy to improve the processability of *i*-P3HB involves its blending with other PHAs, PHA copolymers featuring varied chain lengths, or other polyesters with a similar chemical structure. Such polymers can be obtained biotechnologically, such as poly(3-hydroxybutyrate-*co*-3-hydroxyvalerate) (PHBV) and poly(3-hydroxybutyrate-*co*-4-hydroxybutyrate) (P34HB) [35,36,37,38], or chemically through methods like ring-opening polymerization (ROP) or the polycondensation of hydroxy acids and their esters, as in the case of poly(lactic acid) (PLA) and atactic poly(3-hydroxybutyrate) (*a*-P3HB) [39,40,41,42,43,44].

**Figure 1:**
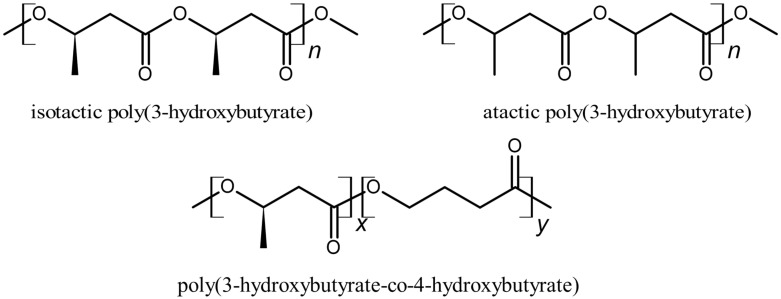
Chemical structure of *i*-P3HB, *a*-P3HB, and P34HB.

While blending *i*-P3HB with polylactic acid (PLA) and [45,46,47] poly(3-hydroxybutyrate-*co*-3-hydroxyvalerate) (PHBV) [48,49,50] has been thoroughly investigated in the literature, blending *i*-P3HB with *a*-P3HB and P34HB has still not been deeply explored. These two polymers—which, next to *i*-P3HB, are the focus of this study—are amorphous and have a similar chemical structure to that of *i*-P3HB. Therefore, it can be expected that they can be blended effectively with *i*-P3HB, resulting in interesting material properties. Figure 1 illustrates the structural differences between *i*-P3HB, *a*-P3HB, and P34HB.

A notable advantage of *i*-P3HB is its resistance to organic solvents, which facilitates its application in different media. However, its limited solubility in organic solvents (mainly soluble in chloroform) has led to the predominance of preparing blends by solution casting using chloroform, which reduces the sustainability of the obtained products.

To address this issue, it would be desirable to blend the polymers through extrusion, a solvent-free process, which, compared to solution casting, is environmentally friendly and compatible with high-throughput manufacturing.

Unfortunately, the extrudability of *i*-P3HB is very limited due to the very small process window, resulting from a melting point of around 175 °C [12,51,52], for the onset of decomposition at a temperature range of 180–190 °C [6], making it difficult to achieve a stable extrusion process [53,54,55,56,57]. The narrow processing window of *i*-P3HB and the thermal degradation during processing present a significant challenge for the application of this biobased and biodegradable polymer. This highlights the urgent need to improve the processability of *i*-P3HB by extending its processing window, thereby avoiding thermal degradation during processing. The incorporation of amorphous *a*-P3HB and P34HB in these blends is expected to circumvent this, by influencing the crystalline phase in terms of lowering the melting point.

While similar blends were obtained in a previous study by solution casting [58], in this study, we aim to explore the potential of the solvent-free blending of *i*-P3HB with *a*-P3HB and P34HB to enhance processability and broaden the application potential of *i*-P3HB-based materials.

## 2. Materials and Methods

Isotactic poly (3-hydroxybutyrate) in powder form with a weight-average molecular weight of *M*w = 260,000 g∙mol^−1^ ± 20,000 g∙mol^−1^ and a polydispersity index of PDI = 2.7 was purchased from Biomer (Schwalbach/Germany). P34HB with *M*w = 196,000 g∙mol^−1^ ± 26,000 g∙mol^−1^, (PDI = 2.3), was purchased from CJ Biomaterials (Seoul, Republic of Korea). *a*-P3HB was prepared by self-polycondensation as described in ref. [59]. *a*-P3HB was produced with titanium isopropoxide as catalyst and had a weight-average molecular weight of 2652 g∙mol^−1^ ± 700 g∙mol^−1^ (PDI = 2.3). Titanium isopropoxide (97%) was purchased from Sigma Aldrich (Darmstadt, Germany). The weight-average molecular weight of the polymers was determined with gel permeation chromatography (GPC) using an Agilent chromatograph (Waldbronn, Germany) conducted in chloroform and calibrated with polystyrene standards. The GPC results were evaluated using the DataApex Clarity Software (version 10.1) (Petrzilkova, Czech Republic).

### 2.1. i-P3HB Blends Preparation by Extrusion

To prepare the blends, binary mixtures of *i*-P3HB with either *a*-P3HB or P34HB were first produced separately, using proportions of 15, 30, and 50 wt.% of the second component. Additionally, binary blends of *i*-P3HB containing 30 wt.% and 50 wt.% of *a*-P3HB were further mixed with P34HB in proportions of 15, 30, and 50 wt.%, having ternary blends containing all three polymers.

Sample labeling was conducted as described in Table 2, by noting the name of the second component in the blend next to *i*-P3HB, followed by their proportion in the blend.

The specified amounts of each polymer were added into a mixing barrel and premixed for five minutes, 30 rpm, and the homogeneity was checked by visual inspection. The mixture was then processed by extrusion using a twin-screw Polylab extruder from Thermo Fisher (Kandel, Germany) equipped with three heating zones and co-rotating screws (16 mm diameter, 640 mm length). The processing temperature and screw rotation speed (rpm) were adjusted for each blend based on the results of the DSC analysis of the same blend obtained by solution casting, as well as observations of the extruder’s torque and the optical homogeneity of the extruded blend.

The specific and optimized processing parameters are listed in Table 3. Once stable processing parameters were achieved, a throughput of 2 kg per hour was maintained for each blend in the laboratory setup.

### 2.2. Preparing of Specimens for Tensile Tests

**Figure 2:**
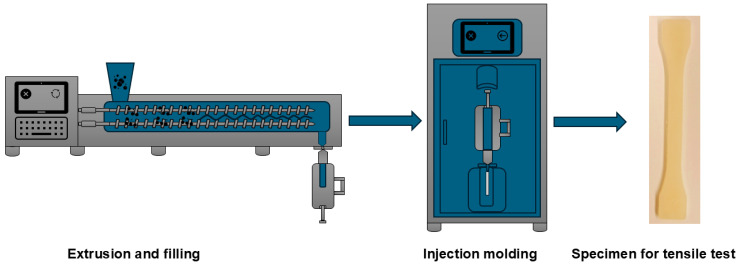
Preparation of specimen for tensile tests by extrusion and injection molding.

Specimens for tensile tests were prepared, as described in Figure 2, in the laboratory of Thermo Fisher (Karlsruhe, Germany). Each blend was plasticized by using the twin screw Minilab extruder, equipped with co-rotating conical screws (110 mm length). To avoid the building of voids, the melt was directly filled in the cylinder of the injection molding machine, HAAKE Minijet, and transferred to injection molding, where tensile specimens (Type 1A) were obtained. The extrusion temperature for all blends was 165 °C, with a screw speed of 60 rpm. The injection molding temperature was also 165 °C, with pressure of 200 bar for 10 s, while the mold temperature was 50 °C. These parameters produced the best results in this process.

### 2.3. Differential Scanning Calorimetry (DSC)

Thermal parameters of the blends, including melting temperature (*T*m), glass transition temperature (*T*g), crystallization temperature (*T*c), their specific enthalpies, and crystalline ratio (*X*c), were determined with a Phoenix F1 204 DSC from NETZSCH (Selb, Germany) under nitrogen flow (20 mL∙min^−1^) and a heating/cooling rate of 10 °C∙min^−1^ from −50 to 200 °C and also from −80 to 50 °C to determine the *T*g. The degree of crystallinity of *i*-P3HB blends was estimated from the melting enthalpy values (∆Hm) of the samples and the melting enthalpy of 100% crystalline *i*-P3HB (∆*H*^0^m = 146 J∙g^−1^) [60] using Equation (1).

(1) $$x_{c}\left[\%\right]=\frac{\Delta H_{m}}{\Delta H_{m\;}^{0}\times w_{i}}\times 100$$

where *w_i_* is the weight fraction of *i*-P3HB in the blends.

### 2.4. Thermogravimetric Analysis (TGA)

To determine the onset of degradation temperature (*T*d) of P3HB blends in terms of mass loss, thermogravimetric analysis was performed with a PerkinElmer TGA 4000 (Rodgau, Germany). The samples (10–30 mg) were heated from room temperature to 600 °C in nitrogen atmosphere (40 mL∙min^−1^) at a heating rate of 10 °C∙min^−1^. The onset of thermal degradation was also calculated using the software Pyris (version 11) of PerkinElmer (Rodgau, Germany).

### 2.5. Gel Permeation Chromatography (GPC)

The weight-average molecular weight (*M*w) of the obtained blends was determined by gel permeation chromatography (GPC) using an Agilent chromatograph (Waldbronn, Germany) conducted in chloroform and calibrated with polystyrene standards. The GPC was equipped with a UV detector and a differential refractive index detector (1260 infinity) with two styrene–divinylbenzene columns (300 mm × 4.6 mm) connected in series with a flowrate of 0.3 mL∙min^−1^ at 40 bar and 30 °C. The samples were prepared by dissolving in chloroform (HPLC grade), with concentration of 5 mg∙ml^−1^, and 20 µL of the solution was injected for the measurement.

### 2.6. Tensile Tests

Tensile tests were conducted according to DIN EN ISO 527-1 [61] at room temperature (23 °C, relative humidity: 50% ± 5%) using a ZwickRoell universal testing machine (Ulm, Germany). For each sample, 5 specimens (Type 1A), which were prepared by injection molding, were tested with a crosshead speed of 5 mm∙min^−1^ and preload force of 1 Newton. The average values and the standard deviations for tensile strength and elongation at break were calculated using the Test Expert Software (version 1.4) of ZwickRoell.

## 3. Results and Discussion

Isotactic P3HB is an attractive candidate to replace petroleum-based plastics, due to its eco-friendliness, biodegradability, and favorable mechanical properties. However, its application is hindered by the brittleness caused by the high crystallinity and difficulties in melt processing, as thermal degradation occurs near its melting temperature. Recent studies aimed to modify *i*-P3HB and improve its processing window employed solution casting using chloroform, which reduces the sustainability of the obtained products [62,63,64,65,66].

Blending *i*-P3HB with *a*-P3HB and P34HB is expected to result in blends that can be processed by extrusion without solvents, at reduced temperatures below the melting point of *i*-P3HB. This is attributed to their amorphous nature, similar chemical structure, and the possible disruption of *i*-P3HB crystallization. Such an approach can extend the processing window of i-P3HB and prevent thermal degradation during processing.

In the following section, the thermal and mechanical properties of *i*-P3HB blends produced by melt extrusion with *a*-P3HB and P34HB will be examined, and their influence on the processing window of *i*-P3HB will be discussed.

### 3.1. Thermal Characterization

***a*-P3HB**: In the first step, *i*-P3HB was mixed with *a*-P3HB to produce binary blends by extrusion. It was expected that *a*-P3HB could improve the processing window of *i*-P3HB due to their similar chemical structures, facilitating extrusion at temperatures below the melting temperature of neat *i*-P3HB to mitigate thermal degradation.

Table 4 presents the thermal properties of *i*-P3HB blends with *a*-P3HB, as determined by DSC. The results of the obtained blends reveal a significantly lower glass transition temperature compared to the neat *i*-P3HB, which has a *T*g of 5.5 °C. Blends with 15 wt.% *a*-P3HB demonstrated a *T*g of −26 °C, while those with 50 wt.% *a*-P3HB demonstrated a *T*g of approximately −42 °C. This reduction indicates that *a*-P3HB acts as a plasticizer, due to its low molecular weight and chemical similarity to *i*-P3HB. This effect enhances the polymer chain mobility, facilitating extrusion processing [67,68,69]. However, similar blends obtained by solution casting in a recent study exhibited stable thermal and mechanical properties even after one month of storage, indicating no notable risk of migration over time [58]. Furthermore, in the present study, the mechanical tests of the obtained blends were carried out after one month of storage to evaluate the potential embrittlement due to post-crystallization.

**Figure 3:**
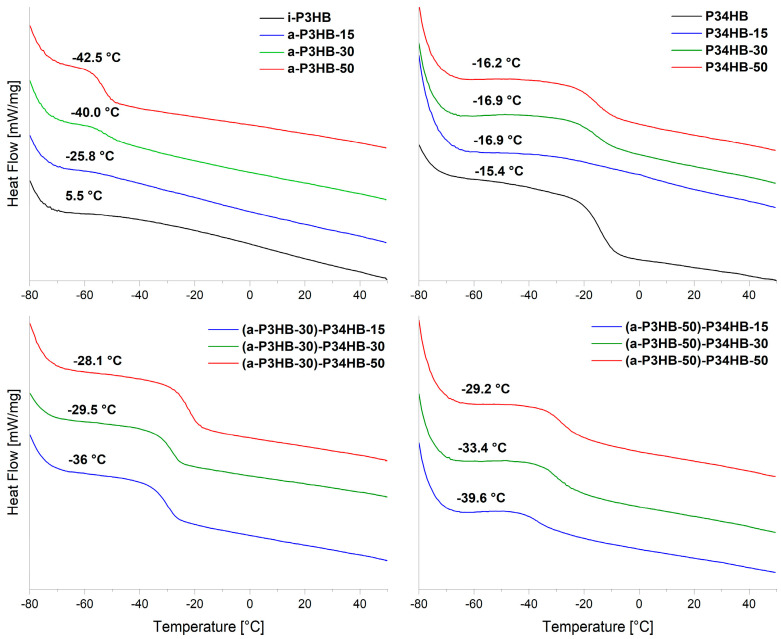
DSC curves (2. heating run) showing the *T*g values of the *i*-P3HB binary and ternary blend.

While DSC curves show only one *T*g, the *T*g of *i*-P3HB may still be present but not detectable due to its high crystallinity (Figure 3). Therefore, further investigations are required to assess the miscibility between the two polymers.

Additionally, blends with 30 and 50 wt.% *a*-P3HB exhibited a notable reduction in the melting temperature of *i*-P3HB, decreasing to approximately 165 °C and 150 °C, respectively. This represents a 10 to 25 °C reduction compared to the neat *i*-P3HB, which has a *T*m of 175 °C. The observed reduction in the *T*m is attributed to the good compatibility between *i*-P3HB and *a*-P3HB, due to their similar chemical structures, which strongly influences the crystallization of *i*-P3HB [70,71,72].

**Figure 4:**
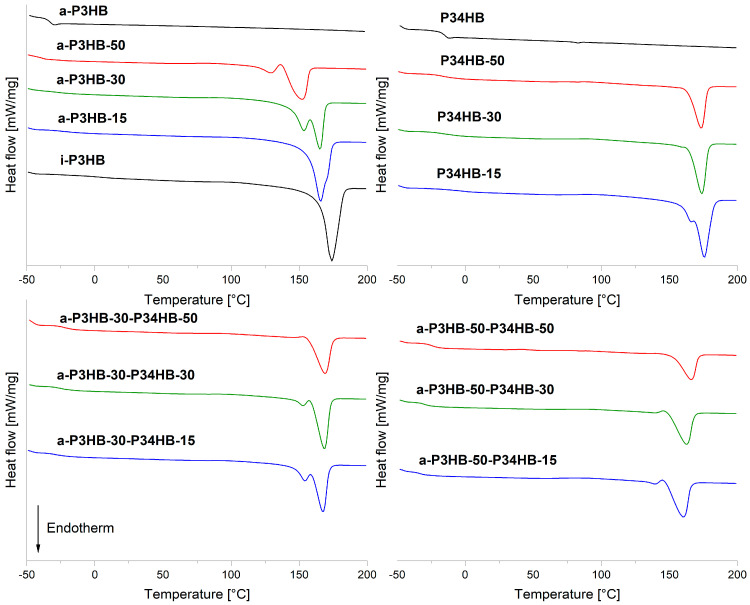
DSC curves (2. heating run) of *i*-P3HB blends.

This change in crystallization behavior is also evident from the appearance of a second melting peak at lower temperatures, indicating the presence of crystals with a different stability caused by the disruption of the *i*-P3HB crystallization (Figure 4) [73]. The impact of *a*-P3HB on the crystallization supports the feasibility of producing *i*-P3HB blends at lower temperatures, thereby reducing the risk of thermal degradation.

The degree of crystallinity also decreased significantly from 68% in the neat *i*-P3HB to 30% in blends with 50 wt.% of *a*-P3HB (Table 4). This reduction is nearly proportional to the weight ratio of *a*-P3HB in the blends. Similarly, the crystallization temperature (*T*c) showed a comparable trend, decreasing to approximately 70 °C in blends with 50 wt.% *a*-P3HB. This shift reflects the increasing ratio of the amorphous polymer, which inhibits the formation of nuclei necessary for crystallization. However, the crystallization temperature can be adjusted by adding nucleating agents to the blends [58,74].

**P34HB**: *i*-P3HB was also mixed with the amorphous copolymer P34HB, and binary blends were produced by extrusion. Due to its amorphous nature and similar structure, P34HB was expected to have similar effects in terms of the crystallization behavior and lowering the melting temperature, thereby improving the processing window of *i*-P3HB.

DSC results of binary blends with P34HB, obtained by extrusion, showed *T*g values at around −16 °C, very close to the *T*g of the neat P34HB (−15.4 °C). This suggests the presence of a second *T*g. However, the DSC curves show only one *T*g, similar to the blends with *a*-P3HB, which can also be attributed to the limited amorphous phase of *i*-P3HB (Figure 3). Therefore, further investigations are required to assess the miscibility of the polymers.

In contrast to the blends with *a*-P3HB, the melting temperature of *i*-P3HB blends with P34HB did not exhibit any depression across all compositions. A second melting peak, appearing as a shoulder, was observed in blends with 15 wt.% P34HB but disappeared as the P34HB content increased (Figure 4). This suggests a limited impact of P34HB on the crystallization of *i*-P3HB, compared with *a*-P3HB (Table 4).

The degree of crystallinity decreased proportionally with the P34HB content, reducing from 68% in the neat *i*-P3HB to 32% with the 50 wt.% P34HB. Similarly, the crystallization temperature showed a reduction, decreasing from approximately 98 °C for the neat *i*-P3HB to about 47 °C with the 50 wt.% P34HB (Table 4). This reduction is due to the increasing proportion of the amorphous polymer in the blend, which shifts the crystallization to lower temperatures.

Since the reduction in crystallinity was observed without any depression in the melting temperature, these blends are considered phase-separated blends. The reduced crystallinity is attributed to the presence of the amorphous P34HB rather than an alteration in the crystallization behavior of *i*-P3HB. The production of these blends by extrusion was only feasible at 175 °C, not, as expected, below the melting temperature of the neat *i*-P3HB. Therefore, no improvement in the processing window of *i*-P3HB was achieved by using only P34HB as the blend component.

***a*-P3HB-P34HB**: Blends containing 30 and 50 wt.% *a*-P3HB were further mixed with P34HB, to produce ternary blends via extrusion. This approach aimed to exploit the depression in the melting temperature and the disruption of the crystallization of *i*-P3HB induced by *a*-P3HB, along with the reduction in crystallinity due to the presence of P34HB. All blends were successfully extruded at temperatures between 150 and 160 °C, which is below the melting temperature of pure *i*-P3HB. This prevented thermal degradation and resulted in optical homogenous melts.

The DSC analysis of the obtained blends revealed that combining *i*-P3HB blends containing 30 and 50 wt.% of *a*-P3HB with P34HB allows for the production of blends with adjustable thermal properties and degrees of crystallinity. The *T*g values of the ternary blends increased with the P34HB content, ranging from approximately −40 to −28 °C. Similarly, the melting temperature of these blends also increased with the content of P34HB, from 160 °C to around 167. This increase in the *T*g and Tm is attributed to the decreasing proportion of *a*-P3HB. Nevertheless, all ternary blends showed significantly lower melting temperatures compared with the neat *i*-P3HB, confirming the improved processability (Table 4).

Among the ternary blends, the *a*-P3HB-50 with 50 wt.% P34HB showed the lowest crystallinity at 21%. This low crystallinity of ternary blends explains why the second melting peak, caused by *a*-P3HB and its disruption of the crystallization, was not detectable. Furthermore, the crystallization temperature *T*c of these blends ranged from 62 to 88 °C, a range that could be further controlled by incorporating nucleating agents as additives.

In summary, the DSC results highlight that blending with *a*-P3HB results in blends with lower melting temperatures and degrees of crystallinity compared to the neat *i*-P3HB (Figure 4). These blends can be processed by extrusion at reduced temperatures, safely below the thermal degradation temperature of *i*-P3HB, which begins at the temperature range of 180–190 °C. In contrast, binary blends with P34HB show reduced crystallinity but maintain the same melting temperature as the neat *i*-P3HB, indicating no improvement in its processing window.

**Figure 5:**
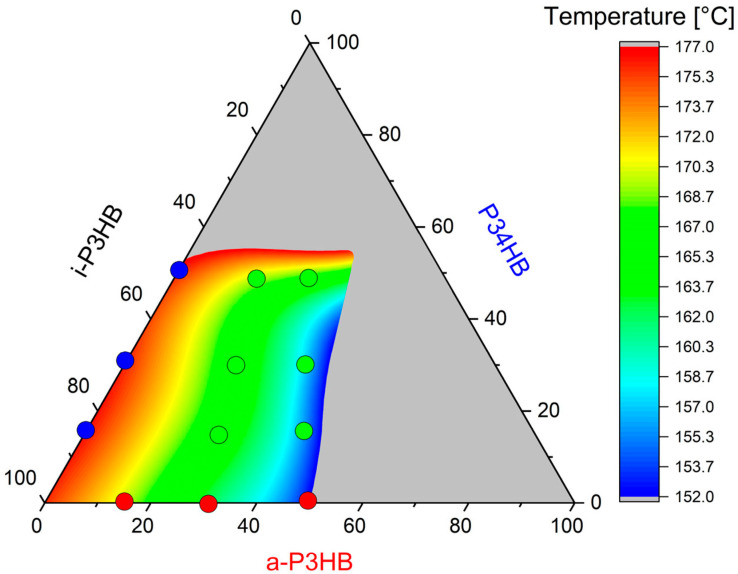
The improvement of the processing window of *i*-P3HB by blending with *a*-P3HB and P34HB.

By combining *a*-P3HB and P34HB with *i*-P3HB, ternary blends with adjustable thermal properties and crystallinity can be obtained. Importantly, these blends can be extruded at temperatures of 150–165 °C, which is 10–25 °C below the melting temperature of pure *i*-P3HB. This facilitates extrusion processing without solvents and avoids thermal degradation. Figure 5 illustrates the extended processing window of *i*-P3HB in a ternary diagram by showing the blends that are processable at reduced temperatures.

The red points in the ternary diagram represent binary blends with *a*-P3HB, which are processable at temperatures between 150 and 165 °C. This is due to the depression of the melting temperature and the disruption of the crystallization induced by *a*-P3HB. In contrast, the blue points indicate binary blends with P34HB, which require higher processing temperatures, as P34HB appears to have no significant effect on the crystallization behavior of *i*-P3HB and its melting behavior. The green points represent ternary blends, which are also processable at reduced temperatures. This is attributed to the combined effects of a depressed melting temperature and reduced crystallinity, allowing for extrusion without requiring high shear rates to achieve a homogeneous melt.

Additionally, the gray zone in the ternary diagram delineates the limits of the proportion of amorphous polymers within the blends necessary to preserve the advantageous mechanical properties of *i*-P3HB and its tunable crystallinity.

### 3.2. Thermal Stability of i-P3HB Blends

The thermal analysis of *i*-P3HB blends showed that blending with *a*-P3HB and P34HB facilitates processing at reduced temperatures of 150–160 °C, which are 15–25 °C below the melting point of *i*-P3HB. To ensure that the extrusion process does not cause the extensive degradation of the molecular weight or affect the thermal stability of *i*-P3HB, both GPC and TGA analyses were performed on the starting polymers and the resulting blends. GPC results provided the weight-average molecular weight (*M*w) of the obtained blends and enabled the assessment of the potential reduction due to the degradation during processing. On the other hand, TGA results indicated the onset of thermal destructive degradation in terms of the mass loss.

**Figure 6:**
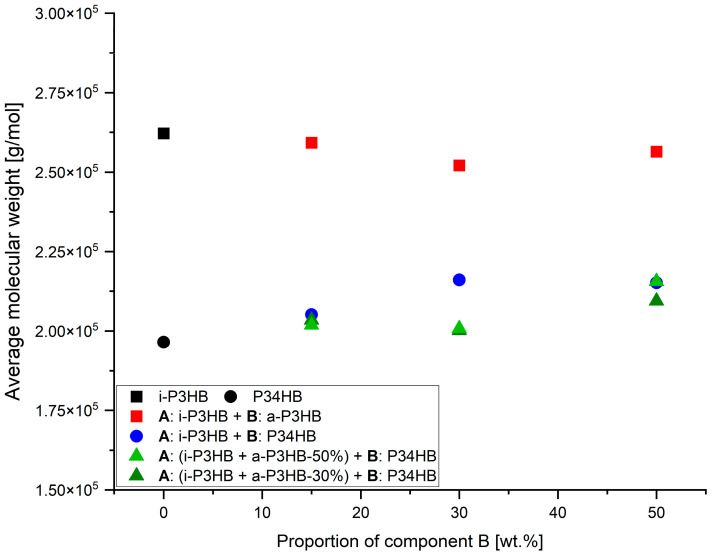
Weight-average molecular weight of *i*-P3HB blends.

Table 5 presents the *M*w values of the *i*-P3HB blends after extrusion, while the *M*w values of the neat *i*-P3HB and P34HB were determined from the unprocessed polymers. The *M*w of blends containing *a*-P3HB ranges between 260,000 and 270,000 g∙mol^−1^, which is very close to that of the *i*-P3HB used in this study. On the other hand, the *M*w of binary and ternary blends containing P34HB ranges between 200,000 and 220,000 g∙mol^−1^. This shift is attributed to the presence of P34HB, which itself has an *M*w within this range (Figure 6). The variation in the *M*w among both binary and ternary blends was approximately 7000 g∙mol^−1^, which is negligible given that GPC is a relative measurement method. This indicates that there was no significant reduction in the *M*w of the polymers during extrusion.

**Figure 7:**
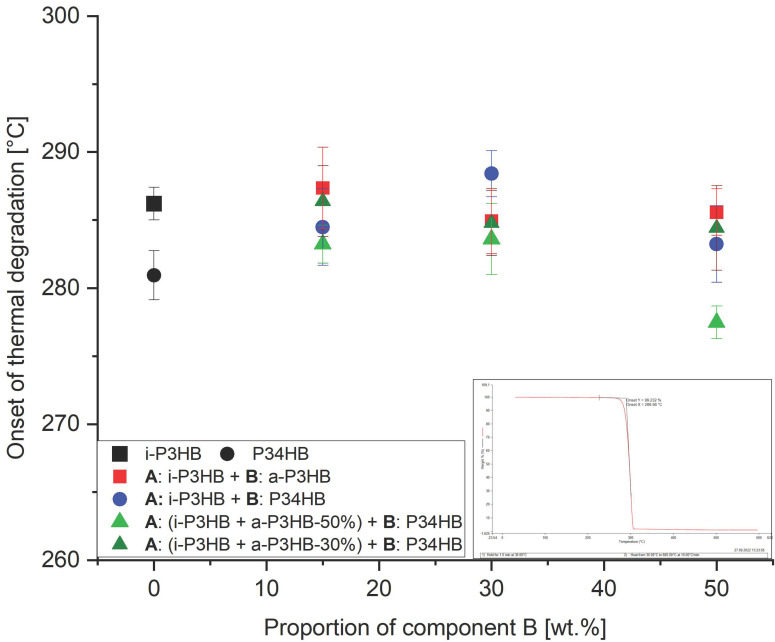
Onset of thermal degradation temperature of *i*-P3HB blends.

While the thermal degradation of *i*-P3HB, in terms of chain scission, starts already at a temperature range of 180–190 °C [6,11], TGA results showed the onset of the thermal destructive degradation (*T*d), in terms of the mass loss, for unprocessed *i*-P3HB and P34HB at 286 °C and 281 °C, respectively (Table 5). Binary blends with *a*-P3HB exhibited *T*d values between 284 °C and 287 °C, which are all closely comparable to that of the neat *i*-P3HB. Similarly, binary blends with P34HB and ternary blends containing all three polymers exhibited *T*d values between 277 °C and 288 °C, which are similarly close to that of the neat *i*-P3HB. These slight differences in the *T*d compared to *i*-P3HB across the samples demonstrate that blending with *a*-P3HB and P34HB does not impact the thermal stability, as the *T*d consistently remained within a narrow range (Figure 7).

### 3.3. Mechanical Characterization

To determine the mechanical properties of *i*-P3HB blends, tensile test specimens were produced via injection molding. Prior to this, the blends underwent plastification through extrusion. However, producing pure *i*-P3HB samples via injection molding was not feasible. The material, in its powder form, melted only at 190 °C, leading to significant inhomogeneities and thermal degradation, as evidenced by strong smoke formation. Additionally, the direct injection molding of *i*-P3HB blends without prior extrusion-based plastification led to inhomogeneities in the specimen, indicating the relevance of prior plastification for further processing.

**Figure 8:**
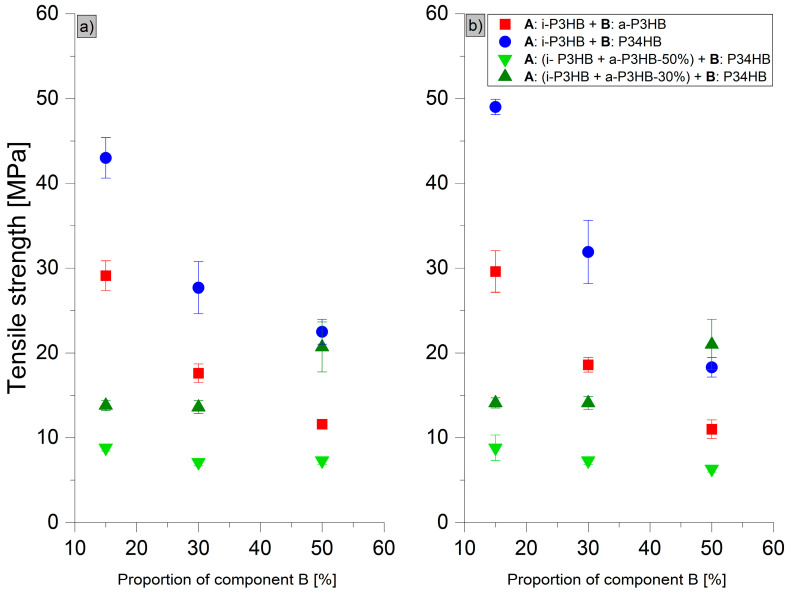
Tensile strength of *i*-P3HB blends (**a**) after production and (**b**) after 30 days of storage.

**Figure 9:**
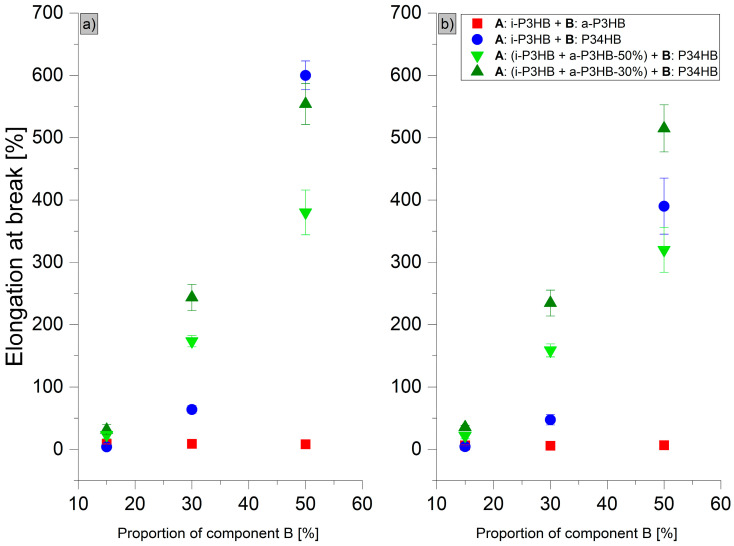
Elongation at break of *i*-P3HB blends (**a**) after production and (**b**) after 30 days of storage.

The results of tensile tests (Table 6) revealed distinct trends in the mechanical behavior of the *i*-P3HB blends. Blends with *a*-P3HB showed no significant improvement in the elongation at break compared to the pure i-P3HB, indicating that *a*-P3HB does not enhance flexibility but primarily influences thermal properties. For example, the elongation at break (*ε*B) of the *a*-P3HB-15 blend was only 8.8%, and even at the higher *a*-P3HB content of 50 wt.%, the *ε*B reached just 8.2%. Over time, these values remained relatively stable, suggesting that the secondary crystallization of *i*-P3HB was hindered. The reduction in the tensile strength in these blends is attributed to the lower degree of crystallinity and the relatively low weight-average molecular weight of *a*-P3HB (Figure 8 and Figure 9).

In contrast, blends with P34HB demonstrated a significant improvement in both the tensile strength and elongation at break, especially at higher P34HB contents. The P34HB-50 blend, for instance, achieved an elongation at break of 600%, which decreased to 390% after one month, indicating a reduction in ductility over time. Despite this decrease, the blend retained a high degree of flexibility. Additionally, the reduction in tensile strength was less pronounced compared to *a*-P3HB blends, due to the higher weight-average molecular weight of P34HB (Figure 8 and Figure 9).

Blends containing both *a*-P3HB and P34HB exhibited a range of tensile strength values (8.8–13 MPa), depending on the polymer’s ratio, and showed an enhanced elongation at break that remained relatively stable over time. For example, the *a*-P3HB-50-P34HB-50 blend achieved an initial *ε*B of 380%, which decreased modestly to 320% after one month, demonstrating a better retention of mechanical properties. Similarly, the blend *a*-P3HB-30-P34HB-50 achieved an initial *ε*B of 528.8%, maintaining a high value of 490% after one month, which is indicative of improved long-term mechanical stability (Figure 8 and Figure 9).

These findings suggest that while *a*-P3HB has a limited impact on flexibility, blending with P34HB at higher concentrations (50 wt.%) enhances the elongation at break but does not preserve the enhanced mechanical properties over time. The combination of *a*-P3HB and P34HB creates a synergistic effect, resulting in a balanced enhancement of the mechanical performance, with an improved elongation at break and stable mechanical properties over time.

## 4. Conclusions

In this study, blends of *i*-P3HB with *a*-P3HB and P34HB were successfully prepared by a solvent-free method through extrusion. The influence of *a*-P3HB and P34HB on the processing behavior of *i*-P3HB was explored, along with an assessment of their thermal and mechanical properties using DSC, GPC, TGA, and tensile tests.

The DSC analysis indicated that the incorporation of *a*-P3HB led to a 15–25 °C decrease in the melting temperature compared to the neat *i*-P3HB and reduced crystallinity. Additionally, the appearance of a secondary melting peak suggested the presence of crystals with varying stabilities. In contrast, the addition of P34HB did not significantly affect the melting temperature but did contribute to a reduction in crystallinity. Ternary blends containing all three polymers exhibited both a lower crystallinity and decreased melting points. This demonstrates that blending *i*-P3HB with *a*-P3HB and P3HB facilitates its processing at reduced temperatures between 150 and 165 °C, thereby avoiding its thermal degradation.

Furthermore, the GPC analysis confirmed that the addition of *a*-P3HB and P34HB did not cause a significant reduction in the weight-average molecular weight of the extruded blends, supporting the conclusion that extrusion within the 150–165 °C range prevents thermal degradation.

In terms of mechanical properties, tensile tests revealed that blending with *a*-P3HB did not improve the elongation at break and slightly reduced the tensile strength. However, mechanical properties remained stable over 30 days, suggesting suppressed post-crystallization. In contrast, binary blends with P34HB exhibited an enhanced elongation at break, reaching up to 600% with 50 wt.% P34HB, although this effect decreased to around 400% after 30 days, probably due to the post-crystallization and lack of compatibility between *i*-P3HB and P34HB, which should be further investigated. Ternary blends of all three polymers demonstrated a tunable elongation at break (ranging from 23% to 529%) and tensile strength (7.2 to 13 MPa), with only minor reductions after 30 days.

In summary, blending *i*-P3HB with *a*-P3HB and P34HB significantly enhances its processability by expanding the processing window and enabling extrusion at reduced temperatures without inducing thermal degradation. The resulting ternary blends exhibit tunable and relatively stable thermal and mechanical properties, making them suitable for various processing techniques such as extrusion, injection molding, and fiber spinning.

This study presents a viable approach to process the biobased and biodegradable *i*-P3HB into blends with properties comparable to conventional plastics, with potential applications in the packaging, medicine, and textile industries.

## Figures and Tables

**Table 1:** Comparison of *i*-P3HB properties with PP/LDPE [12].

	*i*-P3HB	PP	LDPE
Melting temperature [°C]	175	176	110
Glass transition temperature [°C]	4	−10	−30
Crystallinity [%]	70	50	50
E-modulus [GPa]	3.5	1.5	0.2
Tensile strength [MPa]	40	38	10
Elongation at break [%]	5	400	600

**Table 2:** Sample designation and description.

Sample Designation	Description
*i*-P3HB	Neat *i*-P3HB
P34HB	Neat P34HB
*a*-P3HB	Neat *a*-P3HB
*a*-P3HB-15	Blend of i-P3HB with *a*-P3HB in 85:15 weight ratio
*a*-P3HB-30	Blend of i-P3HB with *a*-P3HB in 70:30 weight ratio
*a*-P3HB-50	Blend of *i*-P3HB with *a*-P3HB in 50:50 weight ratio
P34HB-15	Blend of *i*-P3HB with P34HB in 85:15 weight ratio
P34HB-30	Blend of *i*-P3HB with P34HB in 70:30 weight ratio
P34HB-50	Blend of *i*-P3HB with P34HB in 50:50 weight ratio
*a*-P3HB-30-P34HB-15	Blend of *a*-P3HB-30 with P34HB in 85:15 weight ratio
*a*-P3HB-30-P34HB-30	Blend of *a*-P3HB-30 with P34HB in 70:30 weight ratio
*a*-P3HB-30-P34HB-50	Blend of *a*-P3HB-30 with P34HB in 50:50 weight ratio
*a*-P3HB-50-P34HB-15	Blend of *a*-P3HB-50 with P34HB in 85:15 weight ratio
*a*-P3HB-50-P34HB-30	Blend of *a*-P3HB-50 with P34HB in 70:30 weight ratio
*a*-P3HB-50-P34HB-50	Blend of *a*-P3HB-50 with P34HB in 50:50 weight ratio

**Table 3:** Extrusion parameters of *i*-P3HB blends.

Sample	Extrusion Temperature [°C]				Screw Speed [rpm]
	Zone 1	Zone 2	Zone 3	*T*M80 *	
*a*-P3HB-15	70	160	160	165	40
*a*-P3HB-30	70	155	155	160	40
*a*-P3HB-50	70	150	150	155	40
P34HB-15	70	165	170	175	50
P34HB-30	70	165	170	175	50
P34HB-50	70	165	170	175	55
*a*-P3HB-50-P34HB-15	70	155	155	160	40
*a*-P3HB-50-P34HB-30	70	155	150	155	40
*a*-P3HB-50-P34HB-50	70	155	150	155	40
*a*-P3HB-30-P34HB-15	70	155	155	160	40
*a*-P3HB-30-P34HB-30	70	155	150	155	40
*a*-P3HB-30-P34HB-50	70	155	150	155	40

* *T*M80: the temperature of the nozzle.

**Table 4:** DSC results of *i*-P3HB blends.

Sample	*T*g [°C]	*T*m1 [°C]	*T*m2 [°C]	*T*c [°C]	*X*c (%)
P34HB	−15.4 ± 0.4				
*i*-P3HB	5.5 ± 0.3		175.5 ± 1.6	91.7 ± 3.34	68 ± 1.2
*a*-P3HB	−42.4 ± 1.2				
*a*-P3HB-15	−25.8 ± 0.9	166.3 ± 0.4	171 ± 0.1	101.2 ± 1.3	55 ± 0.2
*a*-P3HB-30	−42.1 ± 0.4	153.8 ± 0.2	165.5 ± 0.1	90.2 ± 0.2	48 ± 0.6
*a*-P3HB-50	−42.5 ± 1.3	130.8 ± 1.4	153 ± 0.6	71.4 ± 1.4	32 ± 0.1
P34HB-15	−16.9 ± 0.5	165.6 ± 0.7	175.4 ± 0.4	82 ± 0.6	54 ± 0.6
P34HB-30	−16.9 ± 0.3		173.9 ± 0.1	71.5 ± 1.2	46 ± 0.8
P34HB-50	−16.2 ± 0.1		173.7 ± 0.2	70.4 ± 3.6	32 ± 3.9
*a*-P3HB-50-P34HB-15	−39.6 ± 1.8	138.4 ± 0.2	159.4 ± 0.1	61.7 ± 0.1	29 ± 0.2
*a*-P3HB-50-P34HB-30	−33.4 ± 2.1	140.1 ± 1.1	162.1 ± 0.6	66.7 ± 0.2	25 ± 1.8
*a*-P3HB-50-P34HB-50	−29.2 ± 1.7		165.3 ± 0.5	58.5 ± 0.9	21 ± 0.4
*a*-P3HB-30-P34HB-15	−36.0 ± 1.6	154.4 ± 0.1	167.6 ± 0.1	87.6 ± 0.1	40 ± 0.8
*a*-P3HB-30-P34HB-30	−29.5 ± 1.4	151.7 ± 0.4	167.5 ± 0.2	84.1 ± 0.3	38 ± 0.1
*a*-P3HB-30-P34HB-50	−28.1 ± 0.4		167.7 ± 0.5	78.6 ± 3.3	31 ± 2.4

**Table 5:** GPC and TGA results of *i*-P3HB blends.

Sample	*M*w [g∙mol^−1^]	*T*d [°C]
*i*-P3HB ^1^	262,000 ± 20,000	285.6 ± 1.2
P34HB ^2^	196,000 ± 26,000	280.9 ± 1.8
*a*-P3HB-15	270,000 ± 10,000	287.4 ± 3.0
*a*-P3HB-30	272,000 ± 18,000	284.9 ± 2.4
*a*-P3HB-50	255,000 ± 24,000	285.6 ± 1.7
P34HB-15	205,000 ± 35,000	284.5 ± 2.8
P34HB-30	216,000 ± 41,000	288.4 ± 1.7
P34HB-50	215,000 ± 16,000	283.2 ± 3.5
*a*-P3HB-50-P34HB-15	201,000 ± 25,000	283.2 ± 1.4
*a*-P3HB-50-P34HB-30	200,000 ± 13,000	283.6 ± 2.6
*a*-P3HB-50-P34HB-50	215,000 ± 29,000	277.5 ± 1.2
*a*-P3HB-30-P34HB-15	203,000 ± 30,000	286.4 ± 2.6
*a*-P3HB-30-P34HB-30	200,000 ± 38,000	284.8 ± 2.4
*a*-P3HB-30-P34HB-50	209,000 ± 25,000	284.4 ± 3.1

^1,2^ Neat polymers without processing by extrusion.

**Table 6:** Results of tensile tests of *i*-P3HB blends after production and after 30 days of storage.

	After Extrusion		After 30 Days	
Sample	*σ*B * [MPa]	*ε*B ** [%]	*σ*B [MPa]	*ε*B [%]
*i*-P3HB	40 ^1^	5 ^1^	-	-
*a*-P3HB-15	29.1 ± 1.8	8.8 ± 0.8	29.6 ± 2.4	6.4 ± 1.1
*a*-P3HB-30	17.6 ± 1.1	8.8 ± 2.3	18.6 ± 0.9	5.9 ± 0.5
*a*-P3HB-50	11.6 ± 0.2	8.2 ± 0.6	11.0 ± 1.1	6.6 ± 1.0
P34HB-15	43.0 ± 2.4	4.1 ± 1.0	49.0 ± 0.9	4.3 ± 0.5
P34HB-30	28.7 ± 3.6	23.0 ± 13	32.2 ± 3.4	17.0 ± 6.4
P34HB-50	22.5 ± 1.5	600.0 ± 23	18.3 ± 1.2	390.0 ± 45
*a*-P3HB-50-P34HB-15	8.8 ± 0.4	23.0 ± 5.6	8.8 ± 1.7	8.0 ± 4.5
*a*-P3HB-50-P34HB-30	7.2 ± 0.3	180.0 ± 68	6.9 ± 0.1	120.0 ± 18
*a*-P3HB-50-P34HB-50	7.3 ± 0.4	380.0 ± 36	6.3 ± 0.4	320.0 ± 36
*a*-P3HB-30-P34HB-15	13.0 ± 0.5	26.0 ± 4.2	17.1 ± 0.4	7.9 ± 0.9
*a*-P3HB-30-P34HB-30	12.1 ± 0.7	41.4 ± 20.4	14.2 ± 0.6	15.0 ± 3.2
*a*-P3HB-30-P34HB-50	12.3 ± 0.5	528.8 ± 69.3	11.1 ± 0.6	490.0 ± 51

* *σ*B: tensile strength and ** *ε*B: elongation at break; ^1^ values cited from ref. [12].

## Data Availability

Data is contained within the article.
